# mGem: Toxins from killer plasmids as a universal killing system in the fungal kingdom

**DOI:** 10.1128/mbio.01034-25

**Published:** 2026-08-12

**Authors:** Padraic G. Heneghan, Kenneth H. Wolfe

**Affiliations:** 1 Conway Institute and School of Medicine, University College Dublin, Dublin, Ireland; Instituto Carlos Chagas, Curitiba, Brazil

**Keywords:** fungi, yeasts, virus-like elements, killer toxins, evolution, plasmids

## Abstract

Killer plasmids are cytosolic linear dsDNA plasmids found in many species of Saccharomycotina, the budding yeasts. They encode toxins that give their hosts an advantage against competitor yeasts in the environment. They cause cell death in their prey by targeting non-coding RNAs of the translation apparatus, either tRNAs or rRNAs. We recently discovered that similar toxin systems are integrated into the nuclear genomes of many filamentous ascomycetes. Cytosolic linear plasmids similar to killer plasmids are also present in two deeply divergent phyla of terrestrial fungi (Zoopagomycota and Mucoromycota), although they have not yet been shown to have killing activity. It is becoming apparent that plasmid-encoded toxin systems or toxin-like systems are far more widespread than previously considered and have impacted upon fungal biology and evolution for hundreds of millions of years.

## PERSPECTIVE

Fungal species compete with each other and with other organisms for nutrients, and one of the mechanisms that fungi often use to give themselves an advantage is the production of a toxin (1). Some toxins are small peptides that prevent the predation of fungal fruiting bodies by animals, like the α-amanitin toxin produced by *Amanita* mushrooms (2). Other toxins are “killers” in inter-species and inter-strain competition. One of the most famous killer toxins is zymocin, a tRNA anticodon nuclease produced by a dsDNA virus-like element (VLE) system in the yeast *Kluyveromyces lactis* (3). These VLEs are generally referred to as “linear plasmids.” Recent evidence suggests that they have been associated with fungi for at least 650 million years (4). Their killer toxin systems can be viewed as models for understanding interference competition and for drug development to combat various human and crop diseases (1, 5).

## ZYMOCIN-LIKE TOXINS OF THE BUDDING YEASTS

The budding yeasts (subphylum Saccharomycotina) are unique among fungi because, to date, they are the only ones known to have “killer” activity encoded by an independent, dsDNA linear plasmid in the cytoplasm (1). Linear plasmids with killing activity are called killer plasmids. The best known is pGKL1 of *Kluyveromyces lactis*, which encodes a toxin, called zymocin (6, 7). pGKL1 (~9 kb in size) is always accompanied by a larger “helper” plasmid, pGKL2 (~13 kb), which controls the transcription, translation, and telomere functions of both plasmids (7–10). Similar pairs of killer and helper plasmids are found in some other yeast species. Some plasmids have a mixture of killer and/or helper genes, but without a toxin gene—these are called “cryptic” plasmids (11–13). Overall, linear plasmids are rare, being found in only 1–2% of yeast strains (13, 14).

**Fig 1:**
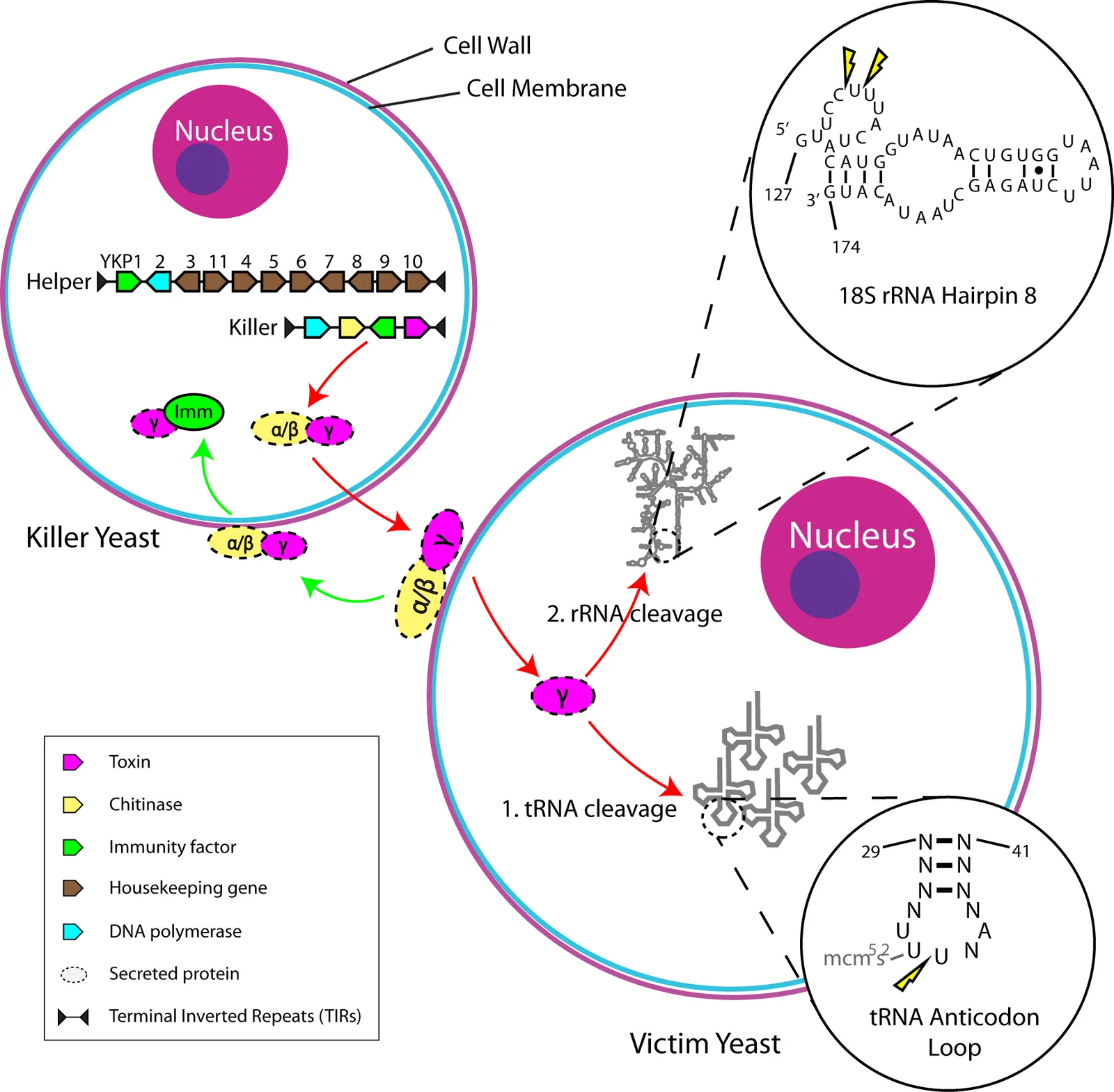
Mechanism of action of zymocin-like killer toxins. The toxin is secreted by the killer cell and enters the victim cell by binding to the chitin of its cell wall (18). Different toxins have two different described mechanisms of action: (1) cleaving tRNA molecules (3, 21) or (2) cleaving rRNA molecules (17). The path of the toxin from the killer yeast to the victim’s targets is shown by red arrows. Lightning bolts indicate the cleavage sites of tRNA and rRNA targets. The toxin may re-enter the killer yeast (green arrows), but the immunity factor also encoded by the killer plasmid binds to and inhibits it (20). A homolog of the immunity factor gene is found on the helper plasmid, but has been shown not to confer resistance to zymocin (22).

Four canonical killer plasmids are known, each of which encodes a toxin (6, 15–17). *K. lactis* zymocin was the first to be characterized. Zymocin is a heterotrimer consisting of α and β subunits formed by cleavage of a chitinase that targets chitin in the cell wall of the victim yeast and a γ-subunit (γ-toxin), which is a ribonuclease (6, 10, 18). The γ subunit is covalently linked to the α/β precursor by a cysteine disulfide bridge and secreted out of the killer yeast to reach the victim (6). After entering the victim cell, zymocin cleaves a specific tRNA molecule, tRNA^Glu^_UUC_, at the wobble position (nucleotides 34–35) of the anticodon loop, causing cell death by stopping translation (Fig. 1) (3). Zymocin is quite reliant on the presence of a modification, 5-methoxycarbonyl-methyl-2-thiol (mcm^5^s^2^), on the wobble uridine (U_34_) for its cleaving activity, and, as a result, the two other yeast tRNAs that contain mcm^5^s^2^-U_34_ (tRNA^Lys^_UUU_ and tRNA^Gln^_UUG_) are secondary targets of zymocin (3, 19). To prevent self-killing, the killer plasmid also encodes an immunity factor, which allosterically inhibits the toxin if it re-enters the host (20).

Zymocin served as the basis for uncovering the targets of three other “zymocin-like” toxins, called DrT, PaT, and PiT, which come from different yeast species. Like zymocin, zymocin-like toxins are encoded on linear plasmids and target non-coding translational RNAs. DrT and PaT are anticodon nucleases that both cleave tRNA^Gln^_UUG_, which is also a secondary target of zymocin. PaT requires an mcm^5^-U_34_ modification for cleavage, whereas DrT does not (16, 23). Unlike the others, the target of PiT is ribosomal RNA. One of its main cleavage sites is a loop in 18S rRNA that bears some resemblance to the anticodon loop of zymocin’s target tRNA^Glu^_UUC_ (17) (Fig. 1).

Because they are encoded by similar plasmids and have similar mechanisms, it has long been suspected that these four toxins evolved from a common ancestor but they share little or no significant amino acid sequence similarity, which makes it difficult to confirm this hypothesis. Recently, bioinformatic mining of budding yeast genome assemblies resulted in the discovery of dozens of additional toxin genes and confirmed that killer plasmids have been associated with the subphylum Saccharomycotina throughout its existence (13). Interestingly, many of the newly discovered zymocin-like toxin genes were found in the nuclear genome, rather than on killer plasmids, apparently as the result of recent integrations of killer plasmids, or parts of them, into their hosts’ genomes. These integrations are probably formed occasionally during repair of dsDNA breaks in the nucleus (24, 25). Many of the newly discovered toxins show killing activity against *Saccharomyces cerevisiae* similar to the canonical toxins, but their molecular targets have yet to be identified. Based on their sequences, there are at least 12 different groups of toxins, and it is possible that all the toxins in a group share the same tRNA or rRNA target (13).

## ZYMOCIN-LIKE TOXINS IN PEZIZOMYCOTINA, THE FILAMENTOUS ASCOMYCETES

It is no surprise that the budding yeasts’ sister subphylum, the Pezizomycotina (filamentous ascomycetes), also produce zymocin-like toxins. However, no killer or helper plasmids have been found in Pezizomycotina. Instead, their toxins are encoded in gene clusters in the nuclear genome. These clusters typically contain three genes: a γ-toxin (ribonuclease), an α/β subunit precursor (chitinase), and an immunity factor (26). These gene clusters probably originated by integration of killer plasmid(s) into the nuclear genome. Unlike those in Saccharomycotina, the nuclear integrations in Pezizomycotina can be inferred to be old because the γ-toxin, chitinase, and immunity genes all contain introns. Introns are a hallmark of nuclear genes in filamentous ascomycetes, whereas linear plasmids never contain introns. The introns must have been gained during millions of years of evolution after the genes arrived in the nucleus.

Pezizomycotina have many chitinase genes, which have been studied extensively because of their role in antagonistic fungal-fungal interactions. They can be divided into three clades: A, B, and C (27). The chitinases in the Pezizomycotina nuclear gene clusters that contain γ-toxin genes belong to subgroup C-II of clade C. They have protein domain structure similarity to the chitinases encoded by Saccharomycotina killer plasmids, and phylogenetic analysis has confirmed that these two groups of chitinases, one nuclear and one plasmid-encoded, are orthologs dating back to the common ancestor of Saccharomycotina and Pezizomycotina (26–29).

Pezizomycotina chitinases are quite modular, with some being fused to an “effector” domain (e.g., an Hce2 domain) (30) that is cytotoxic but unrelated to γ-toxins. However, even Pezizomycotina chitinase genes without an effector domain are upregulated when confronted with competitor fungi (31, 32). It seems likely that the neighboring γ-toxin gene in the gene cluster is also induced, resulting in secretion of a heteromeric toxin in response to competitors. Therefore, the anti-fungal activity attributed to C-II chitinases is probably at least partly due to the action of associated ribonucleases rather than to hydrolysis of chitin alone.

## A KINGDOM OF KILLERS

The origin of zymocin-like killer toxins remains unknown. To shed light on this question, we searched genome sequences from non-Dikarya fungi (i.e., fungi outside the two most familiar fungal phyla, Ascomycota and Basidiomycota). We found linear plasmids in two non-Dikarya fungal phyla, Mucoromycota and Zoopagomycota (4). Some species in these phyla seem to be reservoirs of linear plasmid diversity because they carry multiple different plasmids simultaneously in their cytosol, separate from their nuclear genome—up to 47 plasmids in one isolate. Remarkably, almost every one of these plasmids has its own DNA polymerase gene, implying that each of them replicates independently of the others. A few of the non-Dikarya plasmids contain C-II chitinase genes, but none of them appear to contain γ-toxin (ribonuclease) genes. The function of most of the non-Dikarya plasmids is unclear because they appear not to carry any chitinase, γ-toxin, or housekeeping genes. Nevertheless, we found a phylogenetic signal that the subdivision of linear plasmids into helper types and killer (or killer-like) types is an ancient one, dating back at least 650 million years to the ancestor of terrestrial fungi (4). Given the similarity of their chitinase and DNA polymerase genes to those of Saccharomycotina killer plasmids, we speculated that some non-Dikarya plasmids may be killers that utilize chitinase to transport a different category of toxin (distinct from γ-toxin, and possibly not even a ribonuclease) to their prey.

**Fig 2:**
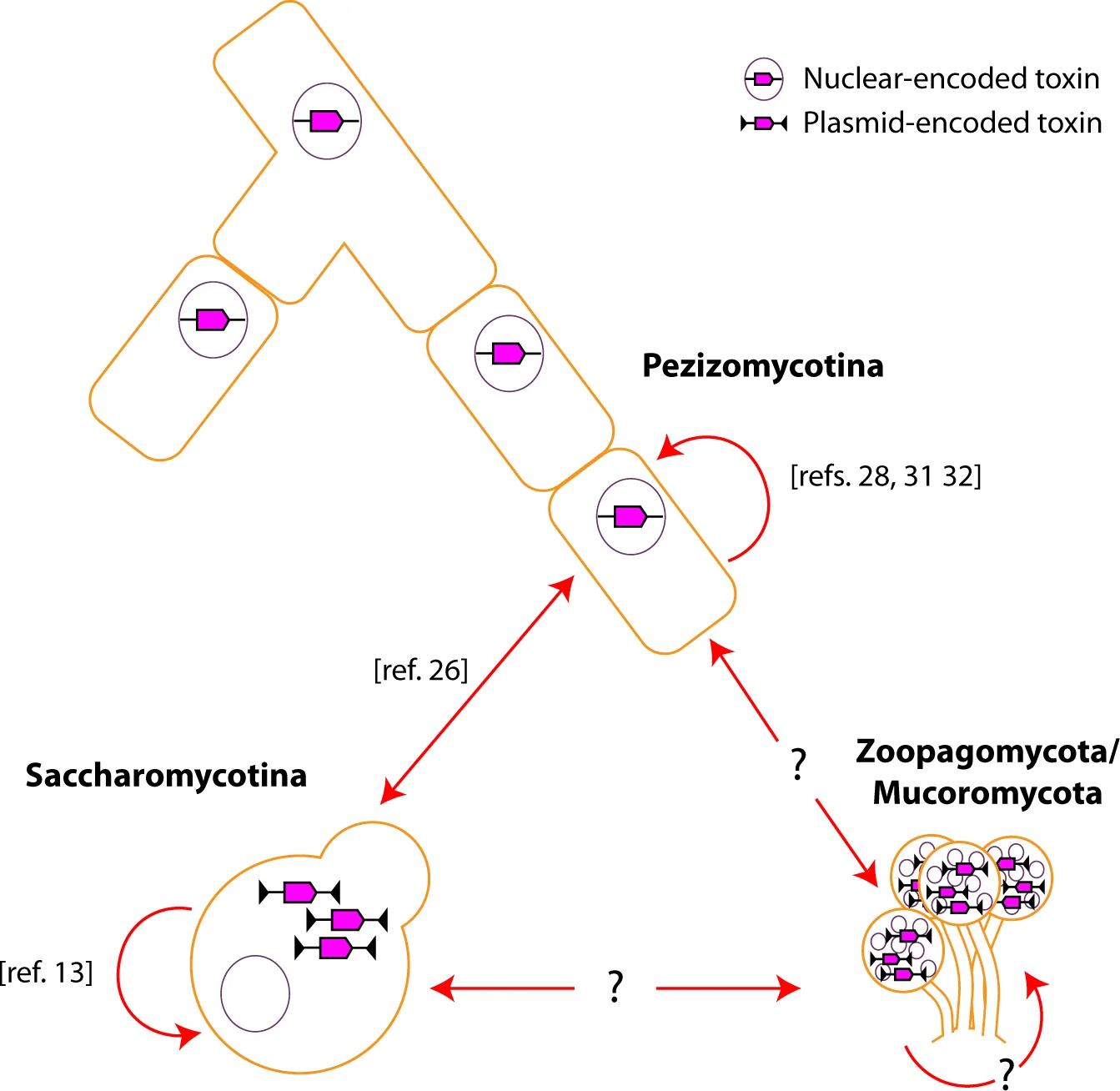
Zymocin-like toxin interactions across the fungal kingdom. Arrows indicate directions of killing activity that have been confirmed or inferred from data on gene expression and gene knockout phenotypes in different species or by assays in *S. cerevisiae*. Question marks indicate interactions that we hypothesize to occur. Literature references for known interactions are shown (13, 26, 28, 31, 32).

The impact of zymocin-like toxins and linear plasmids across the fungal kingdom is extensive. They have been weapons in antagonistic fungal-fungal interactions (Fig. 2), in interspecies wars that have raged for hundreds of millions of years (4). The tRNA-cleaving activity of the toxins may even have been the evolutionary driver of the genetic code changes (reassignment of the codon CUG from Leu to Ser or Ala) that occurred in budding yeasts (33). Knowing this, it is likely that zymocin-like killer toxins could have extensive applications, particularly in agricultural fungi where expansions of chitinase families are often found and attributed to fungal-fungal competition (27, 34). Not only the toxins, but also their immunity factors could be exploited. For example, in the wheat pathogen *Fusarium graminearum*, a toxin gene cluster is one of the fastest evolving loci in the genome (26, 35). A therapeutic target to tackle this destructive pathogen could be developed from the toxin’s immunity factor, leading to the fungus becoming its own worst enemy via self-killing. In the same way, characterizing the functions of unknown genes in linear plasmids from non-Dikarya species has exciting potential to lead to the discovery of new antifungal agents.
